# Face-to-Face with the Doctor Online: Phenomenological Analysis of Patient Experience of Teleconsultation

**DOI:** 10.1007/s10746-022-09652-4

**Published:** 2022-11-28

**Authors:** Māra Grīnfelde

**Affiliations:** University of Latvia Institute of Philosophy and Sociology, Kalpaka boulevard 4 – 322, Riga, LV-1050 Latvia

**Keywords:** Phenomenology, Teleconsultation, Clinical encounter, Qualitative research, Face-to-face encounter, Embodied risk

## Abstract

The global crisis of the COVID-19 pandemic has considerably accelerated the adoption of teleconsultation—a form of consultation between patient and health care professional that occurs via videoconferencing platforms. For this reason, it is important to investigate the way in which this form of interaction modifies the nature of the clinical encounter and the extent to which this modification impacts the healing process. For this purpose, I will refer to insights into the clinical encounter as a face-to-face encounter drawn from the phenomenology of medicine (R. Zaner, K. Toombs, E. Pellegrino). I will also take into account a criticism that has been expressed by various contemporary phenomenologists (H. Dreyfus, T. Fuchs, L. Dolezal, H. Carel), namely, that due to the lack of physical proximity to the other in all types of online encounters, such encounters lack significant features that are present in face-to-face encounters, with the most important of these being the possibility of attaining an empathetic perception of the other and a sense of embodied risk. As these elements are essential features of the clinical encounter, the aim of this paper is to determine whether teleconsultation exhibits these features. To do that, I will integrate phenomenological philosophy with qualitative research drawing materials from both the philosophical tradition, particularly with respect to the concepts of the face-to-face encounter and embodied risk (A. Schutz and H. Dreyfus), and qualitative research study regarding patient experiences of teleconsultation. I will argue that teleconsultation does involve both the possibility of perceiving the other empathetically and the possibility of experiencing a sense of embodied risk.

## Introduction

The global crisis of the COVID-19 pandemic has considerably accelerated the use of teleconsultation (consultation between patient and health care professional via videoconferencing platforms). When in-person, face-to-face consultation began to pose a threat to public safety, many countries followed the recommendations issued by the World Health Organization (WHO), which advocated for the use of telemedicine^1^ to reduce the risk of patients spreading the virus by traveling to hospitals (World Health Organization, 2020). Taking into account this sudden increase in the use of telemedicine, especially in the form of teleconsultation, it becomes important to understand the type of impact that teleconsultation has on the nature of the clinical encounter. While it is clear that video-based, online clinical encounter certainly removes many perceived possibilities for action on the part of both the patient and the health care professional, e.g., the possibility of touching the other person, the extent to which this type of encounter alters the interaction between those parties and the extent to which this modification impacts the healing process (if at all) remain unclear.

This issue becomes especially important in light of the insights into the clinical encounter that can be found in the literature pertaining to the phenomenology of medicine (Pellegrino, 2004; Toombs, 1992, 2019; Zaner, 2006). According to Edmund Pellegrino, at the core of the clinical encounter lies an intersubjective relationship, in which the life-worlds of the patient and the physician meet in order to pursue the concrete goal of healing the patient (Pellegrino, 2004: 194, 196; Toombs, 1992: 89–119).^2^ By reference to ideas found in the work of Alfred Schutz, this relationship is described in the phenomenology of medicine as a face-to-face relationship, namely, as “a relationship in which the participants share time and space, perceiving one another” or as a relationship in which participants are “mutually involved in one another’s biographical situation” by focusing on a common object (Schutz, 1962: 317), i.e., the patient’s experience of illness (Toombs, 1992: 111).^3^ In describing the nature of the clinical encounter, both Pellegrino and Toombs focused on real-life, face-to-face encounters, in which both involved parties are located physically together in the same place at the same time. A significant characteristic of the “face-to-face” encounter, according to Toombs (2019: 223), is the possibility of observing the other person’s bodily expressions, which in turn allows one to perceive the other empathetically, that is, to grasp the other’s experiences as *her* experiences (León & Zahavi, 2016: 228).^4^ This empathetic perception of the other in the context of a face-to-face relationship is of crucial importance to the clinical encounter because in order for this relationship to be successful, it is necessary that each individual in this relationship, whether patient or doctor, “experiences and interprets the other, their respective interpretations of one another, and at the same time within their relationship, experiences and interprets the relationship itself” (Zaner, 2006: 287). While Toombs and Pellegrino did not consider the nature of the *online* clinical encounter, their line of thought rests on two implicit assumptions that might put into question the possibility of empathetic perception in teleconsultation. First, this approach relies on the assumption that expressivity is a necessary condition of the empathetic encounter, and second, it makes the assumption that expressivity is restricted to the presence of the physical body. Real-life, face-to-face encounters offer this physical presence of the other, while online encounters do not.

A great deal of research, both within the phenomenological tradition and outside of it (for example, in sociology and communication theory), has investigated the nature of online interactions. Many thinkers (such as Baym, 2015; Hardesty & Sheredos, 2019; Knorr Cetina, 2014; Maloney, 2013; and Osler, 2020, 2021, to name only a few) have argued that the widespread use of information and communication technologies requires that we rethink traditional views on human interaction as grounded in the face-to-face situation, which is defined by the physical presence of its participants. According to these thinkers, the transformation of the face-to-face domain into a context of mediated interaction offers important social and affective affordances (such as the possibility of feeling empathy, closeness, solidarity, and trust). While these approaches use different theoretical frameworks to develop their arguments, all of them focus on factors other than the presence of the physical body^5^ with respect to explaining the availability of empathy, togetherness, trust and other experiences online. There are also, however, other thinkers who argue for the centrality of the face-to-face situation (and the copresence of physical bodies) in the constitution of intersubjective relationships online. This importance has recently been emphasized by a number of phenomenologists in the context of reflections on the nature of online encounters (Carel, 2020; Dolezal, 2020; Dreyfus, 2009; Fuchs, 2014). Tomas Fuchs (2014) associates online interaction with disembodiment, arguing that the lack of the bodily presence of the other in online contexts leads to the absence of empathetic perception. Luna Dolezal and Havi Carel, two prominent contemporary phenomenologists, are also skeptical of the possibilities for interaction offered by online encounters; however, their positions are less radical than that of Fuchs. Dolezal and Carel claim that online interaction, even online interaction that takes place via video, always compares unfavorably to real-life, face-to-face interaction due to the former’s lack of the immediate presence of the body of the other (Carel, 2020; Dolezal, 2020). According to Dolezal (2020: 23), in online interaction, we can never achieve the same levels of intimacy, closeness and contact that we can achieve in face-to-face interaction. It is important to emphasize in this context that these authors do not argue that empathy is impossible online (as does Fuchs); rather, their claim is that due to the lack of the physical proximity of the other in online interactions, interactions in these situations are always incomplete in some way; for example, they lack any real connections or feelings of closeness to the other person.

Taking into account both that there are different perspectives on this matter and that I am focusing on one specific kind of online encounter, namely, an online clinical encounter via video platforms, I propose to address this issue from the perspective of the patient by relying on the results of my qualitative research study of patient experiences of teleconsultation. In this paper, I therefore focus on investigating whether teleconsultation lacks a significant feature associated with face-to-face encounters, namely, the possibility of the empathetic perception of the other person. In addition, however, I also address the criticism voiced by Carel and Dolezal by determining whether teleconsultation can provide the possibility of closeness to the other. I approach this issue from the perspective of the patient; thus, it must be remembered that the same conclusions might not be applicable to the experiences of the doctor.

Another feature of the face-to-face encounter that is essential to the clinical encounter has been claimed to be lacking in the online encounter, namely, the presence of embodied risk, which refers to feelings of physical and emotional vulnerability under the gaze of the other (Dreyfus, 2009: 69–74). This aspect is essential to any interpersonal encounter because the presence of embodied risk is a necessary condition for ethical relationships. According to Dreyfus, “you have to be in the same room with someone who could physically hurt or publicly humiliate you and observe that they do not do so, in order to trust them and make yourself vulnerable to them in other ways” (Dreyfus, 2009: 69).^6^ Dolezal (2020: 24) agrees, stating that “without physical proximity, embodied risk is drastically attenuated, if not completely eliminated, especially when considering encounters with those we may never have met, or those we do not know well”. In other words, real-life, face-to-face encounters contain this sense of embodied risk, which is a necessary condition for the development of ethical relationships. This requirement is especially relevant in the context of the clinical encounter because of the patient–physician dynamics involved in this context, such that the patient must have trust in the doctor and the doctor must take on responsibility for the patient. Thus, even if teleconsultation offers the possibility of perceiving the doctor empathetically and feeling a sense of closeness to her (as I will argue it does), it remains an important task to determine whether this mode of communication also contains the presence of embodied risk, thereby ensuring the ethical nature of the patient–physician relationship.^7^

The aim of this paper is to determine whether the teleconsultation contains the features of face-to-face encounters that are essential to the clinical encounter, but that have been claimed to be lacking in online encounters, namely, the possibility of empathetic perception of the other and the sense of embodied risk.^8^ I will argue that while it is certainly true that face-to-face interaction is a unique kind of interaction and that this mode of communication can offer unique possibilities for action and interaction, it is not the only kind of interaction that provides the possibility of perceiving the other empathetically and experiencing a sense of embodied risk—teleconsultation can also provide these possibilities. I will prove these claims by integrating phenomenological philosophy with qualitative research. In so doing, I will reference materials from both the philosophical tradition, particularly the concepts of the face-to-face encounter and embodied risk (specifically referring to ideas expressed by Schutz and Dreyfus), and my qualitative research study regarding patient experiences of teleconsultation. As none of the previously mentioned phenomenologists expressing skepticism regarding the possibilities of interaction in online encounters included descriptions of patient’s own experiences of online interaction in their analyses, I believe that remedying this lack might provide a new perspective on the topic.

## Methodology

I approach the issue by integrating phenomenological philosophy with a qualitative study of patient experiences of teleconsultation. This research study involved 14 semi-structured interviews with people who had experienced at least one online video consultation with a medical specialist within the past year.^9^ Among participants, 11 were women and 3 were men, and their ages ranged from 24 to 39 years old. Participants were recruited via several patient organization platforms in [the reference has been taken out for the purposes of the blind review] as well as by using a snowballing approach within the social network of the researcher. Informed consent was discussed with and obtained from all the participants at the beginning of each interview, and all data used in this paper and elsewhere were anonymized. Due to safety restrictions during the COVID-19 pandemic, all interviews took place via the videoconferencing platform Zoom^10^ and lasted between 50 and 90 min each. Interviews were recorded, transcribed verbatim and analyzed with the support of the Nvivo 12 plus program, which facilitated codification.

**Table 1:** Overview of the participants

Number, name	Gender, age	Specialist	Previous in-person familiarity with the specialist	Length of the single consultation	Number of consultations	Videoconferencing platform used
1. Alice	F/37	Internist	No	20 min	1	doxy.me
2. Vilma	F/24	Psychotherapist	No	1 h	6	Skype
3. Andrea	F/39	Otolaryngologist	No	30 min	1	Zoom
4. Thomas	M/31	Neurologist	No	50 min	1	Zoom
5. Dana	F/35	Psychotherapist	Yes	1 h	3	WhatsApp, Zoom
6. Julie	F/28	Gastroenterologist	Yes	20 min	1	Zoom
7. Mark	M/24	Physiotherapist	No	40 min	1	Zoom, MS Teams
8. Sophia	F/26	Internist	No	30 min	1	Skype
9. John	M/35	Psychotherapist	Yes	45 min	10	Zoom
10. Louisa	F/35	Psychotherapist	No	1 h	10	Zoom
11. Agnes	F/33	Psychotherapist	Yes	45 min	6	Skype, WhatsApp
12. Anna	F/37	Family doctor	No	10 min	1	Babylon
13. Maria	F/32	Psychotherapist	Yes	1 h	9	WhatsApp
14. Christina	F/33	Midwife	No	1 h-1,5 h	5	Zoom, WhatsApp

In designing the research study, conducting interviews and analyzing the data, I used the Phenomenologically Grounded Qualitative Research (PGQR) methodology (Køster & Fernandez, 2021) and the “Phenomenological Interview” (PI) framework (Høffding & Martiny, 2016),^11^ both of which argue for the integration of qualitative research with phenomenological philosophy. In so doing, I made use of phenomenological concepts (for example, the concepts of embodiment, affectivity, selfhood) to illuminate the ways in which different dimensions of human existence (as expressed by these concepts) are affected in teleconsultation (see Zahavi & Martiny, 2019: 161). Køster and Fernandez (2021) describe this use of phenomenology’s concepts as a phenomenological grounding of qualitative research, arguing that this grounding allows researchers to focus on specific modifications to certain structural dimensions of human existence. Recently, various research studies have employed one or more core phenomenological concepts (for example, embodiment, intercorporeality, body schema, body image, selfhood, intentionality, affectivity, spatiality and temporality) to ground qualitative research, a phenomenon that has largely, though not exclusively, been seen in the fields of psychopathology and health care (see Klinke et al., 2014, 2015; Slatman, 2016; Yaron et al., 2017; Ekdahl & Ravn, 2021; and García et al., 2021). In this paper, I draw on the concrete structure of human experience expressed by the concept of the “face-to-face relationship” to investigate its particular manifestation in an online environment. In other words, the essential features of the “face-to-face relationship” identified by phenomenologists (such as shared time, shared space and the presence of embodied risk) allow me to focus my qualitative research and to highlight experiential aspects of teleconsultation that could otherwise have been missed.

In the two-tiered fashion proposed by Høffding and Martiny (2016) within the PI framework, I first conducted semi-structured, in-depth interviews to generate nuanced descriptions of patient experiences of teleconsultation and, second, I used these descriptions for phenomenological analysis, with an explicit focus on the face-to-face relationship. The interview process was largely inspired by the framework developed by Høffding and Martiny (2016: 558), who maintain that “in the interview process one should be aware of one’s phenomenological commitments, take up an empathetic, reciprocal and second-person perspective when encountering the subject, and ask specific open questions in order to get descriptions that are as detailed as possible”. Like Køster and Fernandez (2021), Høffding and Martiny argue for the integration of qualitative research—in this case, qualitative interviews—with phenomenological philosophy. According to the latter authors, “the interview is informed by certain phenomenological commitments and in turn informs a phenomenological investigation” (2016: 540).^12^ This study’s interview guide included several predefined focus points structured around categories associated with the concept of the “face-to-face” relationship. For example, participants were asked open questions such as “How did you experience your relationship with the doctor online?,” “How would you describe your contact with the doctor online?,” “How did you feel during the teleconsultation,” “Describe what you could do during teleconsultation?,” and “Describe what you couldn’t do during teleconsultation”. Answers were then explored further through the use of follow-up questions to generate rich and nuanced descriptions of the experiences in question.

The process of analysis was also informed by the study’s phenomenological commitments. The aim of the analysis of these interview descriptions was to uncover the experiential possibilities that are inherent in teleconsultation. In this goal, I agree with Gallagher and Zahavi (2008), who maintain that phenomenology’s goal is not to describe an idiosyncratic experience, but rather to capture the invariant structures of experience (2008: 26).^13^ However, it should be noted that the aim of my research study was not to uncover all the possibilities inherent in teleconsultation. It was to uncover certain experiential possibilities within teleconsultation, which I find to be a relevant task in the context of recent discussions in the literature concerning the nature of the clinical encounter, the possibility of online embodiment and the possibility of online empathy. In addition, the uncovered experiential possibilities should be seen as experiential possibilities and not as conclusive facts about the experience in question.

The process of analysis included three steps. (1) The first step was to bracket from the transcriptions all nonessential material, such as the aspects where participants strayed completely from the topic at hand. (2) The second step was to classify the descriptions of patient experiences of teleconsultation into several categories. It should be noted that these categories were taken from the phenomenological literature; however, they were revised during data analysis. Thus, the generated categories were both theory- and data-driven. Key categories in the context of the whole research project included the following: “embodiment,” “affectivity,” “togetherness with the doctor,” “temporality,” and “spatiality”. (3) The third step was to analyze in further detail the descriptions contained in some of these key categories (in the context of this paper, mainly the categories of “embodiment,”,“affectivity,” and “togetherness with the doctor”) by situating them within the context of phenomenological work that has already been conducted with respect to the nature of face-to-face interaction, the possibility of feeling empathy online and the nature of clinical encounter in general.

## The Possibility of the Empathetic Encounter in Teleconsultation

In this section, I will refer to both the phenomenological tradition regarding the concept of the “face-to-face” interaction and the interview material to determine if it is possible to perceive the other person empathetically in the online clinical encounter. As Zaner, Toombs and Pellegrino have described the nature of the clinical encounter as a face-to-face encounter by reference to ideas expressed in the work of Alfred Schutz, I will also briefly refer to Schutz’s account of the face-to-face relationship. I will highlight the two conditions that are necessary for the face-to-face relationship to occur according to Schutz’s philosophy, and with the help of certain recent interpretations of the concept of empathy, I will argue that these conditions are present in online, video-based encounters as well, thereby allowing for the possibility of the empathetic encounter online. I will also argue that not only empathy but also intimacy or closeness to the other is possible in the online clinical encounter. I will refer to the interview material to support both the claim that the patient can engage in an empathetic encounter with the doctor online and the claim that the patient can feel closeness to the doctor online.

### Necessary Conditions of the “Face-to-Face” Encounter

According to Schutz, the face-to-face relationship essentially involves reciprocal awareness of the presence of another person in temporal and spatial immediacy (Schutz, 1967: 168). He writes: “As I look at you in the community of space and time I have direct evidence that you are oriented to me, that is, that you experience what I say and do […] I know that the same goes for you and that you refer your experiences of me back to what you grasp of my experiences of you” (1976: 30). Thus, two conditions of face-to-face interaction can be distinguished: (1) temporal immediacy and (2) spatial immediacy. In the case of the real-life clinical encounter, as a patient I have a different perspective on or awareness of my illness than that which is accessible to the doctor; however, I am aware that she also has a perspective on my illness and that her perspective is contemporaneous with mine (temporal simultaneity). I am also aware that our perspectives are intertwined in the sense that we influence each other’s experiences. I am aware of all these factors because I perceive expressive movements of the doctor’s body (spatial immediacy). Are these criteria met in the online clinical encounter?

#### Temporal Immediacy

In a live video interaction, the requirement of shared time is met. The live video interaction itself offers the possibility of sharing time—both the patient and the doctor are engaged in a conversation that is occurring to them now. To use Schutz’s (1962: 16) terminology, both patient and doctor grasp each other’s thoughts in a “vivid present”. This fact is evident from the interview material. Participants described the experience of an online consultation as happening ‘now,’ and while some participants did experience technical problems with their internet connection, these difficulties did not influence their experience of sharing the time. This situation can be explained by reference to what participants labeled the ‘new normal of the virtual environment’. Engaging in online communication with the doctor, the patient has certain expectations (e.g., that the screen will freeze from time to time or that issues with sound might occur). These expectations also include the doctor’s response time. Due to these modulated expectations, even if time delays do occur in the context of teleconsultation, the patient can still perceive the doctor’s experience as being part of their shared temporal present (Osler, 2021: 23).

#### Spatial Immediacy

To determine whether it is possible to experience spatial immediacy in an online clinical encounter, it is necessary to examine this concept more closely. Schutz (1967: 163) states that the spatial immediacy of the other person refers to my awareness of her as a present person, namely, as a living, conscious being. I can perceive the other empathetically because I apprehend her body as a field of expression (Ibid, 164). While Schutz (1962: 317) focused on the direct bodily presence of the other person in his descriptions of face-to-face interaction, it has been noted (Hardesty & Sheredos, 2019) that spatial immediacy as described by Schutz refers to the presence of “vivid indications” or “symptoms” of the other’s experiences (Schutz, 1967: 163, 1976: 29). These indications include gestures, gait, facial expressions, intonations and vocal rhythm. (Schutz, 1962: 16). While Schutz made the implicit assumption that expressivity is restricted to the physical body, his account does not stipulate this limitation as a requirement (Hardesty & Sheredos, 2019).

Osler (2021) argues that a live video feed offers the possibility of perceiving the other empathetically precisely because of its ability to grant access to the other’s expressive body.^14^ She refers to the classical phenomenological distinction between the objective, physical body and the lived body, arguing that expressivity need not be restricted to the physical body. The idea underlying this distinction is that there are two main ways in which we experience our body—as an object in the world, from which I can distance myself and which I can examine, and as the feeling and acting subject, which I am. Osler argues that in an empathetic encounter, I experience the other’s lived body and not her object body; more concretely, I perceive the lived body of the other as a field of expressivity (seeing, for example, her reactions to my comments, hearing her tone of voice, etc.). While entering an online space, we do indeed lose the possibility of accessing others’ physical bodies, the lived bodies of others are not lost (2021: 7–11). Schutz (1976: 28) himself notes that concrete, face-to-face relationships differ, i.e., the “symptoms” of the other’s conscious life depend on a concrete situation and vary a great deal. Sometimes the other is accessible to me in an abundance of her bodily expressiveness; however, at other times, these expressions might be severely limited—for example, when a person’s face is only partially visible due to wearing a surgical mask. Osler (2020: 582) points out that we can encounter the other empathetically without having a fully embodied, multisensorial interaction with the other, for example, by encountering the other through a pane of glass (in which case the sense of touch is not present). In another paper, she points out that we would not want to deny that a blind person can empathetically perceive the other through their tone of voice, for example (Osler, 2021: 9). Considering the experience of communicating with a friend on Skype, Osler maintains that even though I am not technically engaging in a direct social relationship, “I do seem to have a direct experience of her expressivity in the sense that it is given to me through her body (as I am attending *to her* and not *to the screen*)” (Osler, 2020: 582).

The interview material supports the claim that it is possible for the patient to perceive the doctor’s experiences directly as *her* experiences while communicating with her online. One participant (Julie) described this in the following way: “I was able to see her [doctor’s] reactions, for example, if she experiences joy. If there were improvements [regarding my health], she instantly became happy and elated”. Another participant (Mark) stated that “At the beginning there were problems with the sound on his [doctor’s] end. He tried to improve the sound and change the background. Then, it was possible to see that the man was confused and irritated”. Other participants noted that they perceived the doctor’s experiences through her expressive body, for example, by perceiving the doctor’s tiredness in the way she sat, seeing her contentment in her smiles, encountering her hesitancy in her posture or recognizing her nervousness in her facial expressions. Many participants claimed that it is very important for them to see their doctor’s bodily expressions to be able to ‘read’ her bodily reactions. Julie describes her experience: “I was focusing on the doctor, I wanted to understand her body language. I wanted to grasp and understand, if she is disappointed in me, because I have done something incorrectly”. This form of perception allows patients to understand the doctor’s intentions and emotions and to receive assurance that the doctor understands them, which although important in any interaction, is of crucial importance in the case of clinical interactions.

### Empathy and Closeness with the Doctor Online

The importance of the doctor’s expressive body can also be illustrated by reference to the moments when the expressive body of the doctor is no longer available. One participant (Vilma) described the possibility of temporarily feeling alone during the teleconsultation due to being unable to see the doctor’s face. She described this situation as follows:

If at some point the light shines directly in the face of the doctor, the camera somehow blocks the ability to maintain eye contact, which leads to a somewhat curious moment of alienation. This is the moment when I feel alone because I am talking to myself. And it is very scary to talk to yourself [laughs]. At least in the sense that a moment ago, I was talking to a human being, but then this contact disappeared (Vilma).

This account indicates that it is possible to experience the doctor as an experiencing subject online (to perceive her empathetically) and that this experience is connected to the possibility of seeing the doctor’s expressive body, i.e., in this case, seeing her face and maintaining eye contact with her.^15^

While this evidence points to the importance of the expressive body (and one might be tempted to think that the more expressivity is present, the more empathy is possible), it should be noted that for some participants, an inability to see the doctor’s face clearly (for example, the doctor might have been located too close to the camera, so that the patient could only see her forehead) did not lead to an inability to experience empathy. This situation can be explained by reference to the fact that for some individuals, for example, individuals with autism, “perceptual access to someone’s expressive body that is not too perceptually rich may well aid empathy, rather than inhibit it” (Osler, 2021: 21). One participant (Maria) described her experience in the following way:

I like that [in the teleconsultation] the contact with the doctor is not so intense [as in the case of on-site consultations], that I can look away and just think and talk, and that I don’t have to endure the actual proximity of the doctor (Maria).

The lack of physical proximity to the doctor as well as limited access to the doctor’s expressive body were actually liberating for some patients—they felt more relaxed and could explain themselves better.

Participants also referred to the importance of reciprocal awareness between them and the doctor during teleconsultation. As Julie describes this aspect, “[i]t was important to hear, understand and see … that the doctor sees that I see, hear and am present”. According to Fuchs (2016: 4), bodily resonance plays an important role in our empathetic encounters. Regarding the affective aspect of such encounters, this resonance amounts to interaffectivity, i.e., “a continuous interaction and mutual modification of both partner’s emotions” (Fuchs, 2014: 157). When we encounter the other empathetically, we simultaneously engage in “a circular, bodily affective communication without even realizing it” (Fuchs, 2014: 157), which leads to the modification of both partners’ emotions. Based on the interview material, mutual modification of both partners’ emotions is also present in teleconsultation. For example, one participant (Thomas) discussed becoming increasingly anxious during teleconsultation due to the doctor’s nervousness, while another participant (Julie) reported having the opposite experience—gradually calming down because she saw that the doctor was very calm.

Interestingly, teleconsultation also offers the possibility for the patient to perceive herself empathetically through the image displayed on the screen (in some video platforms (e.g., Zoom) you see yourself during the consultation) and to modify her own affective states—for example, her emotions. One participant (Vilma) described not only seeing herself on screen during the consultation but also seeing her own emotions (e.g., seeing suffering in her contorted face), which reinforced the emotion in question. Other participants also described experiencing intensified emotions during online clinical consultations (both positive and negative emotions) because they saw themselves experiencing these emotions. Thus, one’s emotions can be modified (i.e., more specifically, intensified) online not only through an encounter with the doctor but also through an encounter with oneself.^16^ To make matters even more complex, one participant (Alice) described the modification of her emotions during teleconsultation in the following way:

It was very interesting that I could see both of us smiling at the same time, because usually when we are in an [on-site] consultation, we cannot see ourselves. But now there are two screens with two smiling human beings and then accordingly, the mood of the communication becomes very positive, because you see both yourself and the smiling doctor, who, well, also makes you smile back. This is like normal mirroring (Alice).

This quotation suggests that the interaffective dynamics operative in this context can involve not only the patient and the doctor or the patient and her own image on the screen but also an interaction among the patient, her own image on the screen and the doctor.

Based on both conceptual analysis of the face-to-face encounter and the interview material, it can be concluded that it is possible to experience empathy in the online clinical encounter. It should be noted, however, that I do not claim that interaction with the doctor online is the same as interaction with the doctor on-site, nor does this evidence suggest that all instances of interaction (both in person and online) offer the same degree of empathy. It is clear that on-site interaction offers different possibilities of interaction than the online meeting, such as, for example, the possibility to touch the other person and receive the touch, smell the other person, smell the room she is in, etc. I have argued that despite these differences, both forms of interaction can offer the possibility of empathetic perception of the other. However, it is possible to talk about the differences in the quality of the empathetic perception. Osler (2021) has suggested to:

think about empathy as something that happens on a spectrum, where I can have a better or worse empathetic grasp of the other – perhaps with simply recognising someone as an embodied subject on one end of the spectrum and empathetically perceiving a close friend and grasping a range of subtle emotions and experiences through their personal style of gestures, tics, expressions, and vitality enriched by my intimate knowledge of them at the other end [..] (24).

Thus far I have argued that teleconsultation offers the possibility to recognize someone as an embodied subject, which could be seen as the basic level of empathy. However, is it possible to have an empathetic grasp of the other in teleconsultation, which would involve more than just the perception of the other as an experiencing subject, opening up the possibility of experiencing connection and closeness with the other? Taking into account the criticism expressed by Dolezal (2020) that online encounters cannot provide the same level of intimacy and closeness with the other as that provided by face-to-face encounters, it is important to determine whether this claim is indeed true. Does the online relationship between the patient and the doctor truly lack the possibility of intimacy and closeness, instead constantly remaining formal and superficial? Before turning to the interview material, it should be pointed out that the quality of one’s empathetic grasp is determined by various factors, such as, for example, the previous knowledge of the other person, the givenness of her expressive body (how rich it is and how much and clearly it is given to me) and the characteristics of the mutual environment. Because these factors vary a lot both within on-site and online interactions, it is possible to claim that empathetic range (and with it the possibility of experiencing contact and closeness with the other) differs both within on-site and online clinical encounter.^17^

Based on the interview material, teleconsultation offers the possibility for the patient to experience closeness to the doctor online. Some participants in the research study maintained that they experienced closeness to the doctor online, which in some cases was even more intense than that experienced during real-life consultations. For example, one participant (Alice) said the following: “I felt closeness in an immediate sense of there being only me and the doctor. And the environment disappears in the sense that there is no going to the medical center, no registration, no waiting in a line, there is only me and the doctor” (Alice).

She continued:

There is a feeling that somehow I can ask questions more freely, that our communication is very unrestrained, not formal at all, and that this is maybe due to the fact that I am not sitting there and looking in doctor’s eyes, I don’t know, but the communication was much freer. (Alice)

Experiential accounts taken from the interviews illustrate the fact that it is possible to experience close interaction with the doctor online despite the lack of the physical proximity of the other. Interestingly, however, these accounts do not highlight the importance of the expressive body as the main constitutive factor of the closeness between the patient and the doctor but rather the characteristics of the online environment. Based on the interview material, two factors can be mentioned regarding the characteristics of the online environment: 1) the lack of a clinical environment, that is, the lack of medical equipment, smells, other personnel coming in and out, patients knocking on doors, etc., and 2) the limited access to the other’s expressive body (only the face of the doctor is visible to the patient and even that can occasionally not be seen clearly). Regarding the former characteristic, the lack of the clinical environment reduces the pressure of social norms and expectations usually associated with the social roles of the doctor and the patient, disrupting to some extent at least the hierarchical relationship between the patient and the doctor and making the interaction between the patient and the doctor less restrained in comparison to in-person clinical interaction. Regarding the latter characteristic, the limited access to the doctor’s expressive body can be liberating for some patients—some patients feel more relaxed and can explain themselves better because they don’t have to focus on the doctor’s body. These two characteristics of the online environment allow the patient and the doctor to focus exclusively on one another (or more concretely, on the mutual problem under discussion), thereby forming a field of intimate co-presence.

The aforementioned impact of the online environment on the constitution of closeness between the patient and the doctor illustrates the effects that digital technology itself has on the constitution of the clinical interaction online. This impact of digital technology on social interaction can be illustrated by reference to Knorr Cetina’s (2014) concept of the “synthetic situation,” i.e., a situation that emerges when social interaction is mediated by screen-based media. A synthetic situation differs from a traditional face-to-face situation in that it makes available to participants something that is spatially and/or temporarily beyond their reach as well as due to the fact that it involves synthetic components, such as the screen itself and “synthetic agents” such as algorithms and software robots. While Knorr Cetina (2014: 48) refers primarily to the example of global financial markets to illustrate the notion of a synthetic situation, videoconference setting also presents a synthetic situation. As such, videoconferencing affords various social and practical possibilities for action and interaction. For example, based on the interview material, videoconferencing technology affords the possibility for the patient to multitask (for example, to search for information on the internet while talking to the doctor) or to hide things from the doctor (for example, by hiding nervous hand gestures or using one’s phone). In the following section of this paper, which is dedicated to embodied risk in the context of teleconsultation, I demonstrate that the screen-based technology associated with videoconferencing also affords the possibility for the patient to obtain increased control over the whole clinical interaction.^18^

So far, I have argued that it is possible to perceive the doctor empathetically as well as to feel closeness to her in the context of teleconsultation. However, this does not mean that there are no limitations inherent in teleconsultation. Some participants referred to the importance of the physical touch of the doctor, which is missing in teleconsultation. This was especially important for patients who were in need of a physical examination. As one participant (Mark), who had back pain, said, “[In teleconsultation] the immediate physical feedback was missing, [the presence of] which would assure me that the doctor has understood me … I think that this diminished the trust in the doctor”. This quotation illustrates the fact that the lack of the physical touch of the doctor in teleconsultation can diminish the patient’s trust in the doctor (I will return to the question about the patient’s trust in the doctor in the next section). The lack of physical touch also points to the increased importance of verbal communication during teleconsultation—in the absence of the physical examination, the patient’s verbal account of her problem becomes very important. This puts a lot of pressure on the patient, who might not be able to give a satisfactory account of her problem.

In addition to this, the online environment comes with some other limitations, which can have an impact on the quality of clinical interaction online. First, the quality of the teleconsultation depends on the successful functioning and mastering of the technology on the part of both involved parties. Second, the online environment offers only limited access to the context of the clinical setting (patient does not see how the doctor interacts with other people, is unable to either see or smell the doctor’s office, etc.), which can provide a richer meaning of the whole clinical situation. Third, the online environment impacts the experience of the consultation by cutting out the transitional space—the patient does not have to go anywhere and sit in the waiting room; she is already there. This lack of the transitional space is seen as something negative by some patients because it takes away the possibility to ‘get yourself emotionally ready’ for the consultation and to calm down after it. Fourth, the quality of the online clinical interaction rests on the ability of the patient to ensure a private place without any disruptions. This was especially difficult for parents with young children present at home—even if the child was in the other room, the focus on the consultation was easily ruptured when the child started to cry.

## Embodied Risk in Teleconsultation

While I have argued that it is possible to perceive the doctor empathetically as well as to feel closeness to her in the context of teleconsultation, it might still be claimed that something important remains missing in this type of interaction. As mentioned in the introduction, Dreyfus (2009) identifies a significant feature of the face-to-face encounter, which, according to him, can never be replicated in online encounters: the presence of embodied risk. Dolezal explains this concept in the following way:

Embodied risk is not just about threats of physical harm (for instance, the threat of physical violence, or in present times, the threat of infection or contamination) but also about the threat of existential or emotional harm. In other words, we can think about embodied risk in terms of one’s social vulnerability (2020: 23).

This emphasis on the importance of embodied risk in the face-to-face relationship is inspired by the work of Emmanuel Levinas concerning the face-to-face encounter as the source of ethical relationships, in which all involved parties are implicated. Levinas (1998) argues that being face-to-face entails an act of self-exposure and a feeling of vulnerability under the gaze of the other. In face-to-face encounters, I am vulnerable to the other since she can hurt me. For this reason, the face-to-face encounter is rife with embodied risk. Apart from physical risk, i.e., the possibility of being harmed physically, there is also existential or emotional risk—I might be “alienated, objectified, scorned, harmed, rebuffed or misunderstood” (Dolezal, 2020: 24). Dreyfus connects this experience of physical and existential vulnerability to feelings of trust. He writes as follows: “(…) it seems that to trust someone you have to make yourself vulnerable to him or her and they have to be vulnerable to you. Part of trust is based on the experience that the other does not take advantage of one’s vulnerability” (2009: 69). He claims that this vulnerability is lost when we are not physically present with one another (2009: 54). In the context of teleconsultation, this criticism is especially important because if teleconsultation truly lacks an ethical dimension, it lacks one of the main constitutive elements of any clinical encounter.

Is it possible for the patient to experience embodied risk online? The findings of the research study show that while teleconsultation does indeed exclude the sense of embodied *physical* risk (the patient cannot be hurt physically online), contrary to the claims of Dreyfus (2009) and Dolezal (2020), it does include the presence of embodied *existential* or *emotional* risk because the patient can be affected emotionally or existentially by the doctor. This possibility to be affected emotionally by the doctor rests on the previously mentioned possibility of perceiving the other empathetically and that of the mutual modification of each other’s emotions. As shown by reference to the concept of interaffectivity, doctor and patient mutually modify each other’s emotions, which can include both positive and negative emotions. The patient can be affected by the doctor both negatively (through the patient being disregarded, misunderstood, judged, shamed, etc.) and positively (through the patient being accepted, listened to, understood, etc.). Interestingly, most participants recounted being affected positively during teleconsultation, especially due to being recognized, understood, acknowledged, and heard. In addition, I believe that a sense of embodied emotional risk is also present in teleconsultation due to the inherently vulnerable position of the patient—when entering a clinical relationship (even in an online form), the patient is already in a dependent position and, for this reason, is in a vulnerable state and can easily be affected by the doctor.

While participants of the research study did not emphasize the existential aspect of the embodied risk, I think that it is possible for the patient to be affected not only emotionally but also existentially by the doctor. Precisely because the doctor has the power to diagnose the patient, that is, she has the power to define the patient, for example, as a person who has a cancer, the very existence of the person with its horizon of possibilities can be impacted. This definitional power, which the health care professional has, can affect the patient not only emotionally (one can become anxious, for example) but also existentially (the meaning that one attributes to one’s life might change). This can happen both in person and online.

It should also be noted that due to the patient’s vulnerable position, the lack of *physical* risk in teleconsultation can work positively, evoking feelings of safety. One participant (Andrea) illustrated this possibility in the following way: “There is safety there; no one can do anything to me against my will. If I won’t do what they want me to do, nothing much can happen to me”. The same participant recalled an incident that occurred in her childhood that directly demonstrates the absence of *physical* risk in online clinical encounters and the positive effect this absence can have on the patient during teleconsultation:

I know that she [the doctor] cannot do anything against my will [during teleconsultation]. I have a childhood trauma. I had pains and tingling in my legs, and my mother took me to the doctor. I think I was 10 years old. And the doctor pulled off my pants and my underpants without any warning. In that moment, I was so shocked that I instantly put my pants back on and ran out of there. I think that I still have this trauma. When I enter the doctor’s office, I am afraid that she will do something to me without any warning (...) [In teleconsultation,] I don’t have to worry that the doctor will pull off my pants. Yes. Her arms don’t reach that far; she cannot do anything to me against my will (Andrea).

The lack of *physical* risk during online clinical encounters is also connected to a sense of control. Another participant (Vilma) expresses this sense in the following way:

The fact that I am behind the screen allows me to feel safe, at least in the sense that at any time I have a power over what will be said, at any time I can mute the doctor, I can take out my earplugs, I can turn away, I can turn off [my computer] if I don’t like something. And this gives me a sense of control over the situation (Vilma).

The lack of embodied *physical* risk is experienced as a positive factor in teleconsultation because it evokes feelings of safety and control on the part of the patient. Taking into account the asymmetrical nature of the clinical relationship, in which the patient is in a vulnerable position, the possibility of being in control that is inherent to online clinical encounters can diminish feelings of vulnerability. Vilma noted this situation as follows: “When I am with the doctor [in real life], I am under her rules and I have to follow them. Video format in some ways allows these relationships to be evened out. Well, in some ways”. These results support the claim made in the previous section of the paper that the screen-based technology associated with videoconferencing affords the patient a sense of control over the clinical interaction.

I have argued that it is possible to experience embodied risk in online clinical encounters (in the form of embodied *emotional* or *existential* risk). Taking into account the fact that Dreyfus and Dolezal connect the experience of embodied risk to feelings of trust, one can conclude that it is possible to experience trust online. This conclusion, however, works only if we accept the premise provided by Dreyfus and Dolezal, namely, that embodied risk is a necessary condition for trusting the other person. While the analysis of this premise exceeds the scope of this paper, it is important to point out that there are alternative views on the issue, emphasizing other factors than the sense of embodied risk, which are necessary for generating trust.^19^ This means that even if the sense of embodied risk is present in teleconsultation, it might still be that the trust is not. Taking this into account, it is important to briefly look at the interview material to determine whether patients experience trust in the doctor online.

While this does not apply to all of the participants of the study, most of them did experience trust in their doctor. It is, however, important to distinguish between patients who had previous in-person interactions with the same doctor and patients who met the doctor for the first time in online consultation. Patients from the first group did not report having any problems trusting the doctor. As long as the trust was already established, it continued to be present online. As one participant (Agnes) said:

We have different roles and masks, but at the core, the person is the same in different environments. In addition, this allowed me to trust the doctor even if [she was online] … It was very important for me that at the core she remained the same.

Patients from the second group, however, had more diverse experiences. Some of the patients who did experience trust in the doctor when meeting her for the first time online talked about the quality of their interaction and the perceived personal investment of the doctor (for example, doctor listening attentively to their problem), which generated trust in the doctor. Other participants referred to the importance of the clinical environment in establishing trust, including the smell and sound of the environment, as well as the formal, visible signs of the doctor as a healer, such as the white coat and diplomas hanging on a wall. One participant (Anna) said.

You see the person for the first time and you decide if you trust her or not … The first impression was very good, very professional. She [the doctor] was dressed very professionally; she had the doctor’s white coat on. In addition, the environment was very professional.

Importantly, precisely because access to the clinical environment is significantly limited in teleconsultation (there is no smell, no waiting room, no diplomas on the wall, etc.) Some participants were unable to experience trust in the doctor. The lack of trust was also experienced by patients who had a distinctly physical problem (for example, back pain) and needed assurance (in the form of the physical touch of the doctor) that the doctor had understood them correctly.

To conclude, this, I have argued that the sense of embodied risk (in the form of *emotional* or *existential* risk) is present in teleconsultation; however, I have also taken into account the possibility that feelings of trust in the doctor online might not depend on the presence of embodied risk. For this reason, with the help of the interview material, I showed that regardless of whether embodied risk is a necessary condition of the experience of trust or not, patients can experience trust in the doctor online.^20^

## Conclusion

Based on the results of the phenomenologically grounded qualitative research study, I argued that teleconsultation contains characteristics that are essential to the clinical encounter, namely, the possibility of empathy and a sense of embodied risk (in the form of embodied *emotional* or *existential* risk). In addition, I demonstrated that, contrary to skepticism regarding the possible quality of online interaction, teleconsultation provides not only the possibility of empathetic perception and a sense of vulnerability on the part of the patient but also the possibility of feeling closeness and contact with the doctor. In addition, online video consultations offer new possibilities for action and interaction that can be useful during the healing process, such as an increased sense of control and feelings of safety on the part of the patient, which transform the traditional, hierarchical patient–physician relationship and are seen as positive factors by some patients. For this reason, one should be cautious regarding the claim that the online form of clinical interaction must always be inferior to on-site, face-to-face interaction. This is not to say that all face-to-face clinical encounters should be substituted with online encounters, whenever possible. There are serious reasons (apart from medical necessity) against taking this approach. For example, people without technological skills and access to technology, as well as those who cannot express themselves verbally, would be excluded from receiving health care. In addition, both the lack of the physical touch of the doctor and the lack of the full embodied perception of the environment on the part of the patient can diminish a patient’s trust in the doctor’s ability to help.

While some of the conclusions that can be drawn from this account might be transferable to other forms of online encounters, first, one must bear in mind the fact that online space is not one homogenous realm, in which only one style of interpersonal relationship is possible. I have referred only to one specific type of encounter, namely, an encounter through a live video feed. Second, I have focused on one particular kind of encounter, namely, the clinical relationship. When I engage in a clinical encounter, I am not only engaging in a relationship with another human being, I am engaging in a relationship with the doctor, while I function as a patient. This type of relationship is deeply impacted by complex forms of personal, professional and social expectations as well as by social norms and institutions (Zaner, 2006: 292). For this reason, the online clinical encounter might exhibit certain characteristics that do not apply to other forms of online encounters.

While it was not the main focus of my paper, the account presented here has implications for phenomenological accounts of any intersubjective interaction, supporting the suggestion made by Lucy Osler (2021) that the presence of the physical body is not a necessary condition for either empathetic relationships (thereby liberating empathy from the context of real-life, face-to-face encounters) or ethical relationships. The account presented here very briefly suggests other sources (apart from the expressive body) that contribute to the quality of the empathetic perception of the other, primarily the online environment itself. More concretely, I have argued that the lack of a clinical environment online, such as the lack of the particular smell, presence of medical equipment, other personnel coming in and out, patients knocking on doors, doctors interacting with other people, etc., reduces the pressure of norms and expectations usually associated with the social roles of the doctor and the patient, making the interaction between the patient and the doctor less restrained in comparison to in-person clinical interaction. Patients experience doctors online not only as doctors (social roles) but also as persons who are easily approachable. While in some cases this weakening of the social roles can reduce trust in the doctor, it can also lead to more open communication and closer contact between the patient and the doctor (which again can increase trust in the doctor). I have also argued that the limited access to the doctor’s expressive body, which is an inherent feature of the online environment (only the face of the doctor is visible to the patient and even that can occasionally not be seen clearly) can also be liberating for some patients—if they don’t have to focus on the doctor’s body, they feel more relaxed and can explain themselves better.

Finally, the account presented in this paper can be situated into the wider discussions about the virtualization of the life world, which takes place through the proliferation of technological devices through which people experience and act within virtual worlds (see, for example, Ollinaho, 2018; Hardesty & Sheredos, 2019). The term virtualization points to the fact that the transformation of communication through technologically based processes (in the context of this paper—videoconferencing platforms) means more than just a mediation of human activity through technology—these processes create novel, virtual realities in which people actively engage into and which can change ways in which people act and interact (Ollinaho, 2018). The results presented in this paper support the idea that the virtual world (at least the virtual world of the clinical encounter) has become a part of the everyday life itself and should not be seen as less real than the sensory perceivable, physical world of concrete objects—patients engage in the interaction with a *real* doctor, namely, with someone who is perceived as an experiencing subject and who has *real* impact on the patient’s life by helping or harming her. Moreover, the results of this research study support both the idea that people are actively engaged in virtual worlds—people are even more actively engaged in online consultations than in on-site consultations (where they usually assume the passive role of the patient)—and the idea that the virtual world can have an impact on the way people act and interact in the everyday world. Regarding the latter, possibilities of interaction offered by the online clinical encounter can illuminate some of the shortcomings of traditional on-site clinical encounters (for example, the power dynamics inherent in the clinical relationship, which comes with the traditional social roles of the patient and the doctor, and which can be detrimental to the healing process) and possibly offer some insights to the health care professionals into how to avoid or at least diminish these shortcomings thereby changing the nature of the clinical encounter as such.

## Footnotes

1 Telemedicine literally means “healing at a distance” and, according to the definition provided by the European Commission (EC), it refers to “the provision of healthcare services where traditional face-to-face patient-doctor interaction (or doctor-doctor) is replaced by over-distance interaction through the use of ICT.” (European Commission, 2019).

2 For both patient and doctor, healing means “making the patient ‘whole’ again, repairing the damage to the bodily or mental integrity, restoring the state of well-being or, if this is impossible, ameliorating the impact of sickness and disease” (Pellegrino, 2004: 196).

3 In this context, illness is distinguished from disease in that the former refers to “perceived lived body disruption” while the latter refers to an “objective pathophysiological finding” (Toombs, 1992: 111–112).

4 In this paper, I will use the concept of empathy as it is typically understood within the phenomenological tradition, namely, as referring to the way in which others’ experiences can become directly perceptually available to me through others’ expressive behavior (see Osler, 2021). In other words, in the context of this paper, empathy is understood as a perception-based experience in which the other’s experience is given to me through her bodily expressivity. It should be noted, however, that empathetic perception is not to be understood as an inferential act—the other’s experience, for example, happiness in her smile, is perceptually given to me ‘directly, unmediated, and non-inferentially’ (Zahavi, 2014: 125). In this paper, I will not refer to the different levels of empathy that have been discussed in the works of certain early phenomenologists, for example, Edith Stein (see Svenaeus, 2021a).

5 For example, Lucy Osler (2021), adopting the perspective of the phenomenological tradition, refers to the presence of the expressive body (versus the object body) as an important constitutive factor associated with social encounters online, while Karin Knorr Cetina (2014), arguing from the perspective of sociology, introduces the concept of the “synthetic situation” (which includes not merely the “response presence” of involved participants but also “synthetic agents” such as algorithms and software robots) to refer to the characteristics of online social situations.

6 This emphasis on the importance of embodied risk in face-to-face relationships is inspired by the work of Emmanuel Levinas concerning the face-to-face encounter as the source of ethical relationships, in which all involved parties are implicated (Levinas, 1998).

7 It is beyond the scope of this paper to evaluate the account offered by Dreyfus and Dolezal on the importance of embodied risk in the formation of trust and ethical relationships. I have, however, chosen to focus on this account both because it is present in recent discussions regarding the nature of online communication and because no one has questioned the claim made by Dreyfus and Dolezal that online interaction lacks embodied risk. I am, however, aware of the fact that there are other, alternative views on the constitution of trust, which use phenomenological tradition as a source of inspiration (see, for example: Brown, 2009; Bizzari, 2022). For this reason, in the section “Embodied risk in teleconsultation” next to arguing that embodied risk is present in teleconsultation, I will also take into account the possibility that embodied risk might not be a necessary condition for developing trust in the other. By doing so, I will look at the interview material, arguing not only that embodied risk is possible in online interaction but also that regardless of whether Dreyfus and Dolezal are correct in their views, it is possible to experience trust in one’s doctor online.

8 It is important to point out at this juncture that within this paper, I consider only one type of online clinical interaction, that is, live, video-based encounters.

9 See Table 1 for an overview of participants, including age, gender, length, and number of teleconsultations, the specialist with whom they consulted, whether they had previous in-person familiarity with that specialist and the videoconferencing platform used.

10 I have chosen to use this particular videoconferencing platform for interviews based on a study comparing the most accessible and most commonly used videoconferencing platforms for qualitative research in terms of criteria such as audio/video recording, one-click access for participants, and privacy features (Lobe et al., 2020).

11 Recently, Høffding and Martiny (together with Roepstorff) (2022) addressed certain objections to the use of phenomenological interviews as a source of reliable and valid knowledge.

12 For more detailed account of how to conduct a phenomenological interview by integrating a qualitative interview with phenomenological philosophy, see Høffding and Martiny (2016).

13 For more detailed account of the differences between phenomenology and introspection, see Gallagher and Zahavi, 2008: 14–28.

14 In his paper on online empathy, Fredrik Svenaeus (2021a) also maintains that live video encounters, such as those conducted via Skype, open up the possibility for an empathetic encounter, since they include “mediations of the lived, expressive human body that is seen and/or heard in the encounter” (92–93).

15 There is an often mentioned problem in the literature on online video communication that there is a lack of eye contact between the communication partners. Friesen explains it in the following way: “You cannot *truly* look an interlocutor in the eye since seeing another’s eyes means looking at the screen. You can give the *appearance* of making eye contact, but this actually requires looking *away* from the other, and into the camera, generally positioned above the screen” (2014: 25). Somewhat surprisingly, while some participants did indeed acknowledge this problem when reflecting about it, they did not experience it as such, that is, most participants said that it was possible to experience direct eye contact with the doctor online.

16 What happens when the patient encounters her own body on the screen? Does she encounter herself as an object body or an expressive body (I am grateful for the anonymous reviewer for raising this question)? The ability to perceive one’s emotions when encountering one’s screen image indicates that it is possible to see oneself on a screen as a field of expressivity or to encounter herself as the lived body. The issue, however, is complex. When one focuses on one’s screen image, it can also provoke an objectification of one’s body. Some participants of the study talked about observing themselves on the screen and experiencing a distance from the observed body. In this case, they encounter themselves as object bodies. This, however, does not necessarily mean that they have lost the perception of themselves as expressive bodies. I think that the distinction introduced by Svenaeus (2021a, 2021b) between “good” and “bad” objectification can be helpful here. He points out that not all objectifications are “bad” ones, leading to the experience of oneself as a mere object, and some of them are “good” ones, retaining one’s sense of subjectivity. The patient can encounter herself as an object body on the screen (by distancing herself from the observed body and reflecting about how it looks, for example) at the same time also seeing her body as a lived or expressive body (by being able to see that she is sad, for example).

17 While the comparison between the empathetic range possible within online interaction and on-site interaction is outside of the scope of this paper, I do not want to maintain that on-site interaction always offers better empathetic perception of the other. It should also be noted that in this paper I am focusing only on a particular kind of online interaction, namely, clinical interaction, which comes with an established forms of conduct, which might be different in other forms of interaction (both online and in person), such as, for example, in the intimate relationship between romantic partners.

18 While the task of investigating the impact of differences in the technologies used for videoconferencing on clinical interactions (for example, by considering the type of videoconferencing platform that is used or whether a smartphone or computer is used to facilitate the videoconferencing) is beyond the scope of this paper, the interview material suggests that these differences do have an impact on the clinical interaction. As one example, the possibility of seeing the image on one’s own screen during teleconsultation (which is a basic setting offered by some videoconferencing platforms but not by others) can afford the possibility of empathetically grasping not only the doctor but also oneself, thus influencing the quality of the clinical interaction.

19 Two examples, which rely on phenomenological tradition, can be given here: Bizzari (2022) in her paper on the experience of online psychotherapy argues that trust is “a felt, bodily openness to the other person,” which is influenced by the “shared, resonant space,” which is primarily bodily in nature (9). Brown (2009), in his paper on trust, uses Shutz’s phenomenology of the lifeworld to argue that it illuminates and explains the primacy of interpersonal communication for the development of trust. According to him, patients “actively construct the interpretive schemes of knowledge on which trust is based” through direct communicative/interactive experiences (403).

20 While the analysis of this issue goes beyond the scope of this paper, there might be other or additional reasons than the sense of embodied risk (*emotional or existential*) influencing the development of the patient’s trust in their doctor online. Further research would be needed to determine how and if at all the sense of embodied risk is connected to factors, which are responsible for generating trust mentioned by the study participants.
